# Phylogeny analysis of complete mitochondrial DNA sequences for pelagic fishes from tuna fishery

**DOI:** 10.1080/23802359.2016.1241675

**Published:** 2016-11-12

**Authors:** Weiwen Li, Shan He, Siquan Tian, Xiaojie Dai

**Affiliations:** a College of Marine Sciences, Shanghai Ocean University, Shanghai, China;; b Scientific Observing and Experimental Station of Oceanic Fisheries Resources and Environment, Ministry of Agriculture, Shanghai, China;; c Key Laboratory of Sustainable Exploitation of Oceanic Fisheries Resources, Ministry of Education, Shanghai, China

**Keywords:** Pelagic fishes, mitochondrial DNA, phylogeny, *Mobula japonica*, *Prionace glauca*

## Abstract

Pelagic fishes captured in the international tuna fisheries have attracted increasing attention in recent years because of declines in their populations. In this study, 58 complete mitochondrial genomes of pelagic species from the classes Teleostean and Chondrichthyans were analyzed. The length of the 58 mtDNA sequences ranged from 15,598 to 18,880 bp, and all of which contained 13 protein-coding genes, 22 tRNA genes, 2 rRNA genes, and 1 control region. Our results suggested that mitochondrial genomes could be a powerful marker for resolving the phylogeny of pelagic fishes. Phylogenetic relationships based on the complete mitochondrial among 58 species indicated that Teleostean and Chondrichthyans are well separated. However, the control region length of *Mobula japonicais* was much larger than the other species in this study. Additionally, the *Prionace glauca* was divided into the clade in the genus *Carcharhinus* which provided a prospective taxonomic status of *P.glauca*.

Tuna fisheries have been prosecuted around the world since at least the pre-1950s (Miyake et al. 2004) and the fisheries brings important economic and social benefits to many nations and areas (Langley et al. 2009; Barclay 2010; Parris 2010). Tuna has a higher economic value compared to other species and the high market demands for tuna have led to significant overcapacity in tuna fishing fleets (Joseph 2003). Furthermore, most stocks of the principal marketable tuna species are nearly fully exploited (Majkowski 2007), as well as the production of bycatch from tuna fishery increases with the fishing fleets. Ecosystem-based fishery management (EBFM) is a new direction for fishery management, essentially reversing the order of management priorities to start with the ecosystem rather than the target species (Pikitch et al. 2004). Management practice proves the EBFM is a more effective management method in the pelagic fishes (Raakjaer et al. 2014; Skern-Mauritzen et al. 2016).

Molecular genetic data has widely been applied for marine fishery management (Waples et al. 2008). The mitochondrial (mt) genome has been widely used as a marker for molecular genetic studies because of its high rate of mutation and exclusively maternal mode of inheritance (Brown et al. 1979; Harrison 1989). Recent studies, however, demonstrated exceptions to these widely held concepts of mitochondrial biology (Ravago et al. 2002). In particular, the observation of mtDNA length variability within species (Rand 1993) challenges the view of evolution, which indicates that more ancient species have with larger genomes.

In this study, the molecular biology of samples from 58 pelagic fishes captured as bycatch in the tuna fishery was used to describe their phylogeny. All the 58 species were accessed from GenBank (Table 1). The mitochondrial DNA length of the 58 species ranged from 15,598 to 18,880 bp. However, the control region of *Mobula japonica* was observed to be as long as 3165 bp, while that of the other species in this study were smaller than 1500bp. The patterns of mitochondrial DNA variation were investigated in pelagic species to examine their evolution.

**Table 1.** Fifty-eight complete mitochondrial DNA pelagic species from tuna fishery.

Species	Accession number	Length
*Xiphias gladius*	NC_012677	16,520
*Tetrapturus audax*	NC_012678	16,526
*T. angustirostris*	NC_012679	16,509
*Makaira mazara*	NC_012680	16,534
*M. indica*	NC_012675	16,526
*Istiophorus platypterus*	NC_012676	16,524
*I. albicans*	NC_022478	16,514
*Thunnus tonggol*	NC_020673	16,528
*T. thynnus*	NC_014052	16,527
*T. obesus*	NC_014059	16,528
*T. maccoyii*	NC_014101	16,527
*T. atlanticus*	NC_025519	16,528
*T. albacares*	NC_014061	16,527
*T. alalunga*	NC_005317	16,527
*Scomberomorus semifasciatus*	NC_021391	16,548
*S. niphonius*	NC_016420	16,646
*S. munroi*	NC_021390	16,796
*S. cavalla*	NC_008109	16,548
*Scomber scombrus*	NC_006398	16,560
*S. japonicas*	NC_013723	16,568
*S. australasicus*	NC_013725	16,570
*Rastrelliger kanagurta*	NC_019624	16,537
*R. brachysoma*	NC_013485	16,539
*Katsuwonus pelamis*	NC_005316	16,515
*Gymnosarda unicolor*	NC_022496	16,510
*Gasterochisma melampus*	HQ425781	16,506
*Euthynnus alletteratus*	NC_004530	16,520
*E. affinis*	NC_025934	16,513
*Auxis thazard*	NC_005318	16,506
*A. rochei*	NC_005313	16,501
*M. japonica*	NC_018784	18,880
*Sphyrna zygaena*	NC_025778	16,731
*S. lewini*	NC_022679	16,726
*Triaenodon obesus*	NC_026287	16,700
*P. glauca*	NC_022819	16,705
*Carcharhinus plumbeus*	NC_024596	16,706
*C. melanopterus*	NC_024284	16,706
*C. longimanus*	NC_025520	16,706
*C. leucas*	NC_023522	16,704
*Rhincodon typus*	NC_023455	16,875
*Megachasma pelagios*	NC_021442	16,694
*Isurus paucus*	NC_024101	16,704
*I. oxyrinchus*	NC_022691	16,701
*Carcharodon carcharias*	NC_022415	16,744
*Cetorhinus maximus*	NC_023266	16,670
*Alopias superciliosus*	NC_021443	16,719
*A. pelagicus*	NC_022822	16,692
*Pseudocarcharias kamoharai*	NC_026216	16,694
*Ranzania laevis*	NC_007887	16,478
*Mola mola*	NC_005836	16,488
*Masturus lanceolatus*	NC_005837	16,481
*Lagocephalus lagocephalus*	NC_015343	16,442
*Assurger anzac*	NC_022494	16,510
*Ruvettus pretiosus*	NC_022493	16,202
*Rexea solandri*	NC_023952	16,350
*Seriola lalandi*	NC_016869	16,532
*Grammistes sexlineatus*	NC_024108	16,502
*Lampris guttatus*	NC_003165	15,598

A phylogenetic tree was constructed based on the complete mitochondrial DNA of 58 pelagic species from tuna fishery (Figure 1) to understand the evolutionary relationship and the position of pelagic species. Maximum-likelihood fits of 24 different nucleotide substitution models (Table 2) were tested before constructing the phylogenetic tree; the results showed that TN93 + G + I model fits these data the best for the neighbour-joining tree (NJ Tree). The NJ Tree was conducted in MEGA 5 (MEGA Inc., Englewood, NJ; Tamura et al. 2011) with bootstrap values of 1000 replicates. The NJ Tree successfully divided the Teleostean and Chondrichthyans into two separate groups, and all the genus species were well determined except for the *Carcharhinus* and *Prionace*. The results of phylogenetic tree indicated that *M. japonica* is a more ancient species, since it stands at the root of the tree. The length of mitochondrial DNA of *M. japonica* was much larger (Table 1) than that of the other species in this study which provided further evidence that *M. japonica* is a more ancient species in the pelagic species.

**Figure 1.**
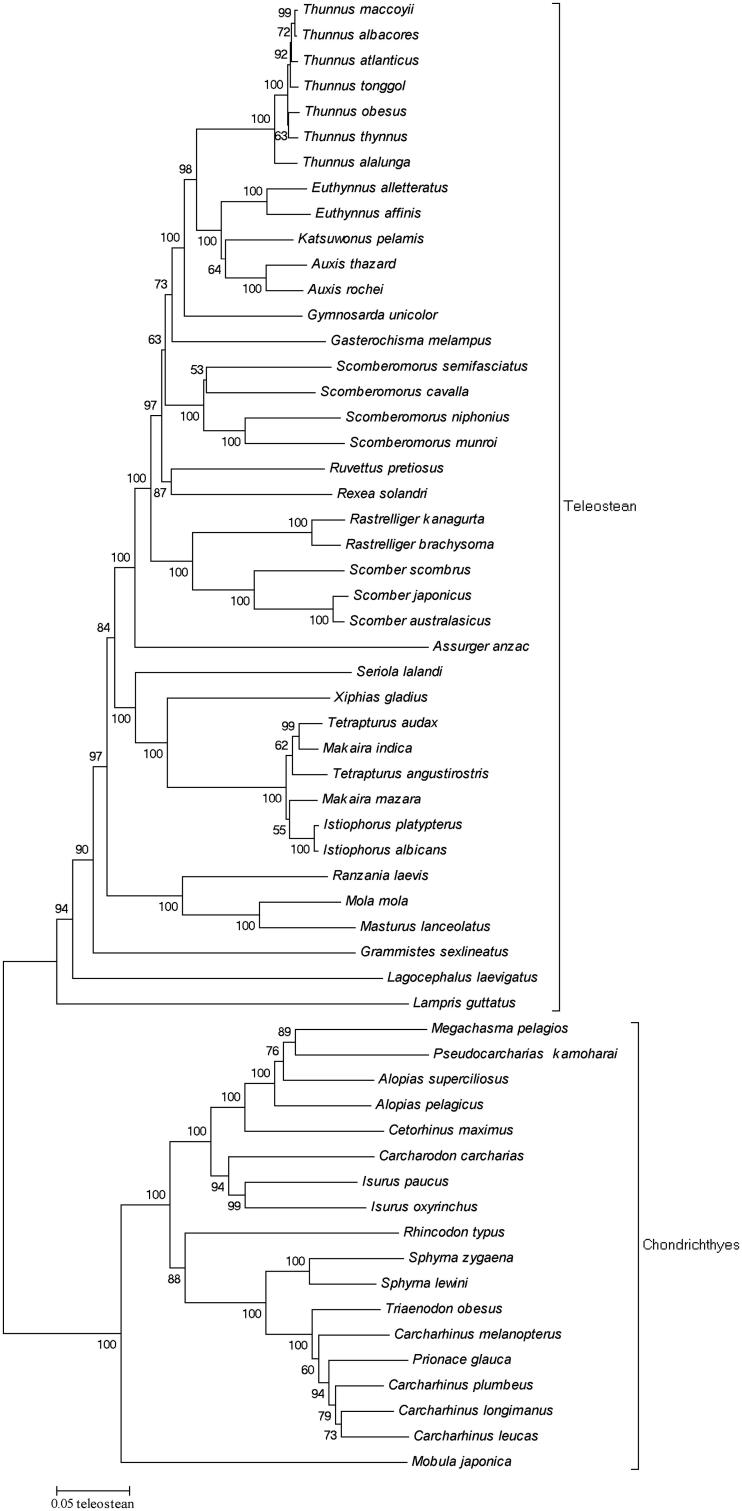
Neighbour-joining tree of 58 pelagic species from tuna fishery.

**Table 2.** Maximum-Likelihood fits of 24 different nucleotide substitution models.

	Parameters	BIC	AICc	lnL
TN93 + G + I	120	**310,914.5**	309,579	−154,669
HKY + G+I	119	311,048.6	309,724.2	−154,743
TN93 + G	119	311,932.7	310,608.4	−155,185
HKY + G	118	312,093.4	310,780.1	−155,272
GTR + G+I	123	315,473.4	314,104.5	−156,929
T92 + G + I	117	316,506.8	315,204.7	−157,485
GTR + G	122	316,521.1	315,163.4	−157,460
T92 + G	116	317,512.9	316,222	−157,995
K2 + G + I	116	317,604.1	316,313.1	−158,041
K2 + G	115	318,626.4	317,346.5	−158,558
TN93 + I	119	326,781.9	325,457.6	−162,610
HKY + I	118	327,024.2	325,710.9	−162,737
GTR + I	122	328,570.8	327,213	−163,484
T92 + I	116	330,908.6	329,617.6	−164,693
K2 + I	115	331,949.2	330,669.3	−165,220
JC + G+I	115	341,733.1	340,453.3	−170,112
JC + G	114	342,696.7	341,427.9	−170,600
JC + I	114	353,594.4	352,325.7	−176,049
TN93	118	364,714.3	363,401	−181,582
GTR	121	365,086.2	363,739.6	−181,749
HKY	117	365,107.1	363,805	−181,785
T92	115	366,807.8	365,527.9	−182,649
K2	114	367,965.7	366,697	−183,234
JC	113	388,505.1	387,247.5	−193,511

Models with the lowest BIC scores (Bayesian Information Criterion) are considered to describe the substitution pattern the best (shown in bold font). For each model, AICc value (Akaike Information Criterion, corrected), Maximum Likelihood value (lnL), and the number of parameters (including branch lengths) are also presented (Nei & Kumar 2000). GTR: General Time Reversible; HKY: Hasegawa-Kishino-Yano; TN93: Tamura-Nei; T92: Tamura 3-parameter; K2: Kimura 2-parameter; JC: Jukes–Cantor. Non-uniformity of evolutionary rates among sites may be modelled by using a discrete Gamma distribution (+G) with 5 rate categories and by assuming that a certain fraction of sites are evolutionarily invariable (+I). Whenever applicable, estimates of gamma-shape parameter and/or the estimated fraction of invariant sites are shown. Assumed or estimated values of transition/transversion bias (*R*) are shown for each model, as well. They are followed by nucleotide frequencies (*f*) and rates of base substitutions (*r*) for each nucleotide pair. Relative values of instantaneous *r* should be considered when evaluating them. For simplicity, sum of *r* values is made equal to 1 for each model.

*Prionace glauca* with different morphologic characters from *Carcharhinus*, such as long pectoral fins greater than internarial, a first dorsal fin origin well behind the rear angle of the pectoral fin, dermal gill rakers, bigger upper tooth with jaws, and much fewer rows of teeth than that of *Carcharhinus* (Compagno 1988). Moreover, a prolonged and gradual process of clasper of *P. glauca* making it rather difficult to be used for determining the maturation of males in *Prioncae* (Compagno 1984). Furthermore, the colour at the top of *P. glauca* is dark indigo blue, while generally brown, blue bronze, and olive in the species of *Carcharhinus*. But, Naylor’s allozyme electrophoretic analyses (Naylor 1989) indicated that *P. glauca* was included in the gene of *Carcharhinus*. And Dosay-Akbulut (2008) used the ribosomal IT1-2 regions sequences to present the phylogenetic relationship with the genus *Carcharhinus*, the bootstrap tree of the NJ (Kimura) indicated that *P. glauca* is a member of genus *Carcharhinus*. A bootstrap tree of NJ was constructed with the whole mtDNA sequences from tuna fisheries species in this study, which point out that *P. glauca* was cladded into the group of genus *Carcharhinus*. Consequently, the NJ Tree based on the whole mtDNA sequences can be considered a powerful marker for resolving the phylogeny relationships within pelagic species.
